# Biochemical and Comparative Proteomic Analyses Delineate the Anti‐Ovarian Carcinogenic Roles of Modified Calycosin

**DOI:** 10.1002/fsn3.71338

**Published:** 2026-01-14

**Authors:** Fuhong Yang, Xin Li, Hanchi Gao, Pei Yao, Xian Qin, Xiao Lin, Keng Po Lai, Jing Tian, Jian Chen

**Affiliations:** ^1^ School of Basic Medical Sciences Guilin Medical University Guilin China; ^2^ Guangxi Health Commission Key Laboratory of Tumor Immunology and Receptor‐Targeted Drug Basic Research Guilin Medical University Guilin Guangxi China; ^3^ State Key Laboratory of Marine Environmental Health City University of Hong Kong Hong Kong, SAR China; ^4^ Department of Psychiatry Icahn School of Medicine at Mount Sinai New York USA; ^5^ Department of Applied Science, School of Science and Technology Hong Kong Metropolitan University Hong Kong, SAR China

**Keywords:** calycosin, cell apoptosis, cell cycle, ferroptosis, ovarian cancer, proteome

## Abstract

Ovarian cancer, the eighth leading cause of cancer‐related deaths globally, is projected to result in approximately 307,000 deaths by 2040. So, identifying novel therapeutic compounds is critical to improving the survival rate of patients with ovarian cancer. Calycosin, derived from Astragalus root, has demonstrated anti‐cancer properties, suggesting its possible use for treating ovarian cancer. In the present study, we synthesized and evaluated a series of calycosin derivatives (H1–H10) to enhance its therapeutic efficacy against ovarian cancer. Among these, calycosin derivative H10 exhibited the most potent anti‐cancer activity, effectively inhibiting cell proliferation, migration, and colony formation abilities in SKOV3 and A2780 ovarian cancer cell lines. In addition, H10 induced G0/G1 cell cycle arrest and dose‐dependent apoptosis in these cells. Further, comparative proteomic analysis coupled with Ingenuity Pathway Analysis was used to delineate the molecular mechanisms underlying the anti‐ovarian cancer effect. Our results demonstrated that H10 modulated key biological processes related to DNA damage response, chromatin and kinase activities, ferroptosis, FoxO signaling, and p53 signaling in ovarian carcinoma. Specifically, H10 regulated a protein cluster comprising RAD51AP1, USP1, USP22, DDX11, ACSL4, GPX4, NCOA4, CCNB1, and CDK1, which are critical to ovarian tumorigenicity. Functional assays confirmed H10's ability to induce cell cycle arrest, senescence, and apoptosis, while proteomic analysis further highlighted its regulatory role in cell cycle regulation and ferroptosis. These findings identify calycosin H10 as a promising therapeutic candidate for ovarian cancer, offering novel insights into its molecular mechanisms of action.

## Graphical abstract

The modified calycosin derivative H10 was more effective in inhibiting cell proliferation through G0/G1 cell cycle arrest, together with inhibiting the migration and colony formation abilities of ovarian cancer cell lines SKOV3 and A2780. Comparative proteomic analysis coupled with Ingenuity Pathway Analysis further delineated calycosin derivative H10‐controlled biological processes related to DNA damage response, chromatin and kinase activities, ferroptosis, FoxO signaling, and p53 signaling in ovarian carcinoma.

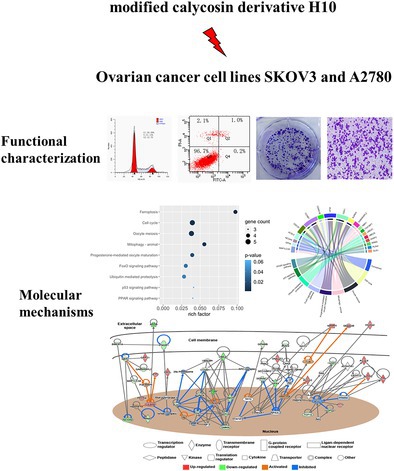

## 1 Introduction

According to GLOBALCAN data, ovarian cancer was the eighth leading cause of cancer incidence and deaths worldwide. It was estimated that in 2020, there were approximately 313,959 new cases of ovarian cancer and 207,252 deaths from ovarian cancer globally (Cabasag et al. 2022). The incidence rates of ovarian cancer have been generally decreasing over time in high‐income countries, but increasing in low‐income countries (Webb and Jordan 2017). It is predicted that the number of new ovarian cancer cases and deaths worldwide will reach 428,000 and 307,000, respectively, by 2040, with low HDI countries seeing increases of 96% and 100% in new ovarian cancer cases and deaths, respectively, to that of 2020 levels (Cabasag et al. 2022). Ovarian cancer cases detected early on during local‐stage disease have a relatively high 5‐year survival rate at 93%, but serous carcinomas detected during stage III and stage IV of the disease had much lower 5‐year survival rates, at 42% and 26%, respectively (Torre et al. 2018). So, the identification of a novel anti‐ovarian cancer drug is urgently needed.

Calycosin is an isoflavonoid compound and the major bioactive component in dry root extracts of Astragalus (*Radix Astragali*), a traditional Chinese medicinal herb (Gao et al. 2014). It is the major determinant of the pharmacological properties of the Chinese herbal decoction Danggui Buxue Tang, including its estrogenic, erythropoietic, and osteogenic activities (Gong et al. 2015). Calycosin has also been found to have other pharmacological effects, including anti‐inflammatory, antioxidant, and antitumor activities (Gong et al. 2021). Various pharmacological activities of calycosin and its derivatives have been reported, such as anti‐oxidant activity. For example, calycosin‐7‐O‐β‐D‐glucoside attenuated ischemia–reperfusion‐induced oxidative damage in HT22 neuronal cells via reduced MDA and ROS levels and increased SOD activity (Yan et al. 2019). Also, calycosin protected against α‐synuclein amyloid‐induced oxidative stress by reducing ROS levels and recovering GSH content and CAT and SOD activities (Pan et al. 2021). In another study, calycosin exhibited antioxidant activity in the skeletal muscle of a chronic kidney disease rat model, including increased SOD and CAT activities and reduced MDA levels, as well as altered levels of proteins in the AMPK/SKP2/CARM1 signaling pathway (Hu et al. 2020). In addition, the anti‐inflammatory potential of calycosin has been studied. For example, calycosin was reported to inhibit diabetes‐induced renal inflammation by reduced expression of the inflammatory cytokines TNF‐α and IL‐1β in cultured mouse tubular epithelial cells, as well as reduced phosphorylation of IKBα and NF‐κB p65 in the kidneys of diabetic mice (Zhang et al. 2019). In an osteoarthritis mouse model, calycosin reduced the expression of inflammation mediators including iNOS and COX‐2 in the cartilage, which was associated with improved cartilage morphology, including increased numbers of collagen II‐ and aggrecan‐positive chondrocytes. Calycosin also attenuated IL‐1β‐induced inflammation in mouse chondrocytes in vitro by reducing expression of iNOS, COX‐2, IL‐6, and TNF‐α, as well as inhibiting the PI3K/Akt and NF‐κB pathways (Shi et al. 2022). Also, calycosin was shown to suppress the methylprednisolone‐induced inflammation in rats through decreased serum levels of TNF‐α, IL‐1β, and IL‐6, and also reduced protein levels of TLR4 and NF‐κB in the femoral head (Zhu et al. 2021).

A number of studies have reported on the anti‐cancer effects of calycosin. For example, calycosin inhibited the proliferation, invasion, and migration rates of both T47D and MCF‐7 breast cancer cells by inhibiting the expression of basic leucine zipper ATF‐like transcription factor (BATF), resulting in downregulated TGFβ1 signaling (Zhang, Lin, et al. 2021). The effect of calycosin on inhibiting breast cancer cell proliferation was also shown to be mediated through overexpression of the long non‐coding RNA WDR7‐7, which activates the G‐protein coupled estrogen receptor 30 (GPR30) (Tian et al. 2017), or through ERβ‐induced inhibition of IGF‐1R (Chen et al. 2014), whereas migration and invasion were inhibited via suppressing Rab27B‐dependent signaling (Wu et al. 2019). In AGS gastric cancer cells, apoptosis was induced in response to calycosin, mediated through modulation of the MAPK/STAT3/NF‐κB signaling pathways, which was associated with increased ROS levels. Calycosin also induced cell cycle arrest at the G0/G1 phase and inhibited the migration of AGS cells, with reduced expression of the migration‐related proteins SNAI 1, E‐cadherin, and β‐catenin (Zhang, Zhang, et al. 2021). Similar effects were observed in HepG2 hepatocellular carcinoma cells; calycosin induced apoptosis through activation of the MAPK, STAT3, and NF‐κB signaling pathways, inhibited migration via decreased expression of TGF‐β1, SMAD2/3, SLUG, and vimentin, and also induced cell cycle arrest at the G0/G1 phase (Liu et al. 2021). Calycosin also exerts anti‐cancer effects through ERβ‐mediated signaling. For example, calycosin induced apoptosis in MG‐63 osteosarcoma cells via inhibition of PI3K/Akt signaling, and the effect was reversed by the ERβ inhibitor PHTPP (Tian et al. 2020). Also, calycosin induced apoptosis and inhibited cell proliferation of several colorectal cancer cell lines including SW480, HCT116, and LoVo in an ERβ‐dependent manner, which was associated with inhibition of IGF‐1R and PI3K/Akt signaling pathways and also suppressed tumor growth in xenograft mouse models (Zhao et al. 2016; Zhu et al. 2022).

More importantly, limited reports have demonstrated the anti‐tumor activity of calycosin and its derivatives against ovarian cancer cells in vitro. For example, calycosin treatment inhibited cell proliferation and induced apoptosis in SKOV3 ovarian cancer cells in a dose‐dependent manner, which was mediated through the upregulation of the Bax/Bcl‐2 ratio and increased levels of cleaved caspase‐3 and cleaved caspase‐9 (Zhou et al. 2015). Also, calycosin‐7‐O‐β‐D‐glucoside augmented the effect of cisplatin on apoptosis of SKOV3 cells, with enhanced modulation of the apoptotic proteins p53, caspase‐3, caspase‐9, Bax, and Bcl‐2 (Huang et al. 2022). In the present study, we modified the chemical structure of calycosin to synthesize more efficient calycosin derivatives. By using the in vitro ovarian cancer models including A2780 and SKOV3 cell lines, we aim to study the induced efficiency of new calycosin derivatives for treating ovarian cancer. In addition, we apply comparative proteomic analysis to delineate the mechanisms underlying the anti‐ovarian cancer role of new calycosin derivatives. Our results showed that the modified calycosin derivatives H10 had lower LC50 in ovarian cancer cells. Moreover, the proteomic analysis highlighted the control of a cluster of proteins involved in DNA damage response, chromatin and kinase activities, ferroptosis, FoxO signaling, and p53 signaling of ovarian cancer cells, suggesting a potential novel drug for treating ovarian cancer.

## 2 Materials and Methods

### 2.1 Synthesis of Calycosin Derivatives

0.5 g of calycosin, 0.21 g of 4‐Dimethylaminopyridine (DMAP), and 0.36 g of triethylamine were added to 20 mL acetone in a round bottom flask with stirring. Then, haloalane was slowly added to the mixture with heat reflux at 40°C, reacting to the end point (TLC monitoring). The acetone was spin‐dried and sequentially extracted with 20 mL H_2_O three times, 20 mL saturated Na_2_CO_3_, and 30 mL saturated NaCl solution. Then, the extract was dried with MgSO_4_ under reduced pressure to remove the solvent. The residue was purified by column chromatography {eluent: V (petroleum ether): V (dichloromethane) = 1:1} to give a yellow solid H1‐H10 (Falasca et al. 2025). The structure of the target compound was confirmed by 1H NMR, 13C NMR, and IR. The obtained calycosin derivatives (H1‐H10) were dissolved in DMSO for the follow‐up experiments.

### 2.2 Cell Culture

The ovarian cancer cell lines A2780 and SKOV3 were purchased from Shanghai Cell Resource Center, Chinese Academy of Sciences. The SKOV3 and A2780 were incubated with McCoy's 5a medium and RPMI 1640 medium, supplemented with 0.5% penicillin–streptomycin and 10% fetal bovine serum (FBS) under 5% CO_2_ at 37°C, respectively. The cells were treated with different concentrations of calycosin derivatives (0, 3, 6, and 12 μM) for 48 h. After the treatment the cells were subjected for cell proliferation, cell apoptosis, cell migration, immunostaining, and proteomic analysis.

### 2.3 CCK8 Assay

The cell proliferation was determined by using EnoGeneCell Counting kit‐8 (CCK‐8) activity kit (Liang et al. 2025). Briefly, 5000 cells were seeded in a 96‐well plate. After 24 h, the cells were treated with different concentrations of calycosin derivatives (0–12 μM). After 48 h of treatment, 10 μL of CCK‐8 solution was added to each well and incubated for 4 h. The value of absorbance (OD) at 450 nm was determined by a microplate reader.

### 2.4 Cell Cycle Assay

The cell cycle was assessed by using the Cell Cycle and Apoptosis Detection Kit. Briefly, 80,000 cells were seeded in a 6‐well plate. After 24 h, cells were treated with calycosin H10 (0–12 μM) for 48 h. Then the cells were harvested and stained with propidium iodide at 37°C in the dark for 30 min. The cell cycle was measured by using flow cytometry with red fluorescence at the excitation wavelength of 488 nm and was used to analyze cellular DNA content.

### 2.5 Cloning Formation Assay

700 cells were plated in a 6‐well plate. The cells were treated with calycosin H10 (0–12 μM) for 48 h. After the treatment, the cell colony was allowed to form for 14 days. Then, the cells were fixed with 4% poly‐formaldehyde for 20 min, followed by 0.1% crystal violet staining for 30 min. Finally, the colony was observed under microscopy, and the picture was captured (Liang et al. 2025).

### 2.6 Cell Apoptosis Assay

The cell apoptosis was assessed by using the Annexin V‐FITC/PI apoptosis double staining kit (Liang et al. 2025). 80,000 cells were seeded in a 6‐well plate. After 24 h, the cells were treated with calycosin H10 (0–12 μM) for 48 h. Then the cells were harvested and resuspended in 300 μL of Binding Buffer. 5 μL of Annexin V‐FITC reagent and 10 μL of propidium iodide (PI) reagent were added to the cells and kept at 37°C in the dark for 30 min. The stained cells were analyzed using flow cytometry.

### 2.7 Wound Healing

2 × 10^5^ cells were seeded in a 6‐well plate. After 24 h, a scratch was applied to the cell monolayer with a 10 μL gun head. Then the cells were washed with PBS to remove the floating cells, followed by replacement with fresh medium containing 1% FBS. The cells were treated with calycosin H10 (0–12 μM). After 24 and 48 h, the cells were photographed, the scratch healing rate was calculated (Liang et al. 2025).

### 2.8 Transwell Assay

The invasiveness of the cells was assessed by using a Transwell chamber coated with Matrigel matrix (Liang et al. 2025). The cells were diluted in the ratio of Matrigel matrix: serum‐free medium = 1:8. A total of 100 μL diluted Matrigel matrix was evenly spread on the bottom of the chamber and incubated in a 37°C incubator for 3 h. 1 × 10^4^ cells were seeded into the chamber. The chamber was placed into a 24‐well plate, and 500 μL medium containing 10% FBS was added to the lower chamber. After 24 h, the cells were treated with calycosin H10 (0–12 μM) for 48 h. Then, the chamber was removed and the cells on the chamber surface were gently wiped with a cotton swab. The cells were fixed with 4% paraformaldehyde fixative for 30 min. The chamber was dipped once with PBS and stained with 0.1% crystal violet for 30 min. After the staining, the chamber was washed three times with PBS and dried. The chambers were viewed under an inverted microscope and photographed at high magnification (200×).

### 2.9 Proteomics Analysis

The SKOV3 cells were seeded in a Petri dish. After 24 h, the cells were treated with DMSO and 12 μM calycosin H10 for 48 h. Then, the cells were harvested for comparative proteomic analysis by using the service provided by Lianchuan Bio Inc. (Wang et al. 2016). Briefly, the cells were lysed in SDT buffer, followed by sonication and boiling for 15 min. After centrifugation, the supernatant was measured by using the BCA Protein Assay Kit (P0012, Beyotime). 200 μg of proteins were incorporated into 30 μL SDT buffer (4% SDS, 100 mM DTT, 150 mM Tris–HCl pH 8.0). The solution was purified by using UA buffer (8 M Urea, 150 mM Tris–HCl pH 8.5) and repeated ultrafiltration (Sartorius, 30 kD). Then, the protein suspensions were digested with 4 μg trypsin (Promega), and 100 μg peptide was subjected to TMT labeling according to the manufacturer's instructions (Thermo Fisher Scientific). The labeled peptides were fractionated by RP chromatography using the Agilent 1260 infinity II HPLC system with XBridge Peptide BEH C18 Column. LC–MS/MS analysis was performed on a Q Exactive Plus mass spectrometer (Thermo Fisher Scientific) coupling with Easy nLC (Thermo Fisher Scientific).

## 3 Results

### 3.1 Anti‐Tumorigenic Roles of Calycosin H10 in Ovarian Carcinoma

**FIGURE 1:**
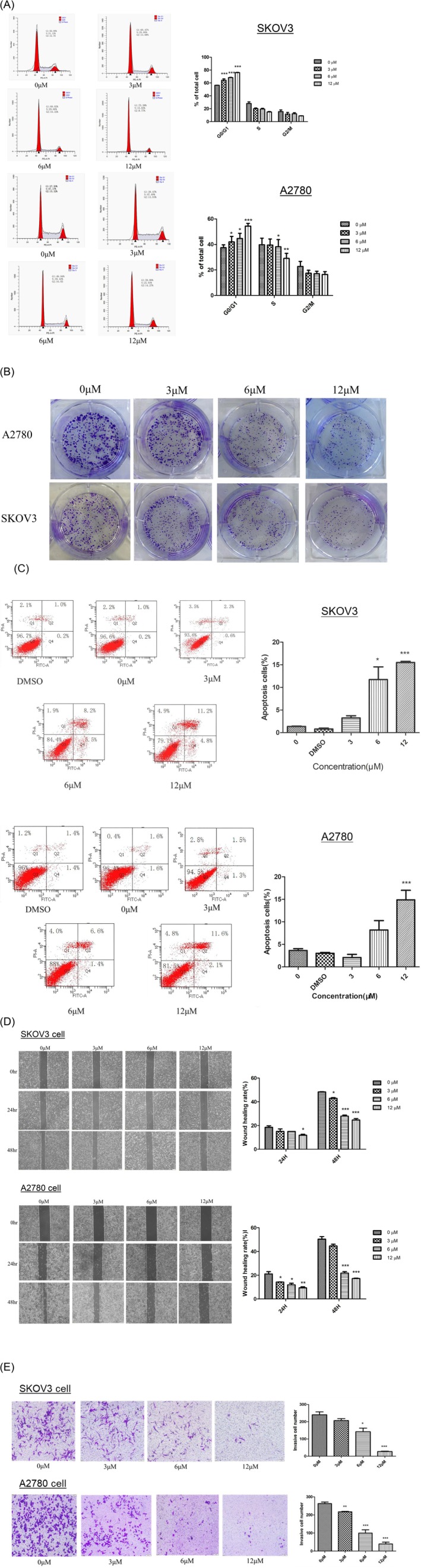
Treatment of calycosin H10 reduced the tumorigenicity of ovarian carcinoma. (A) Propidium iodide staining followed by flow cytometry analysis showed that calycosin H10 caused dose‐dependent cell cycle arrest at the G0/G1 phase in SKOV3 (upper panel) and A2780 (lower panel). (B) Colony formation assay demonstrated the dose‐dependent reduction in the number of colonies caused by the calycosin H10 treatment. (C) AnnexinV‐PI staining followed by flow cytometry analysis showed the induced cell apoptosis in SKOV3 (upper panel) and A2780 (lower panel) caused by the calycosin H10 treatment. (D) Wound healing assay demonstrated the reduced migration ability in SKOV3 (upper panel) and A2780 (lower panel) caused by the calycosin H10 treatment. (E) Invasion assay using matrix gel chamber showed the reduced invasion ability in SKOV3 (upper panel) and A2780 (lower panel) caused by the calycosin H10 treatment.

First, we synthesized a series of calycosin derivatives named from H1 to H10 by structural modification of the calycosin (Figure S1). The lethal concentration (LC_50_) of modified calycosin for inhibiting the growth of ovarian carcinoma (SKOV3 and A2780 ovarian cancer cell lines) was determined by using CCK8 assay. Our results showed that the A2780 cell was more sensitive to most of the modified calycosin (H4, H5, H8, H9, and H10), but SKOV3 only responded well to calycosin H10 (Table 1). Then, we investigated the cell cycle arrest in ovarian carcinoma caused by calycosin H10 treatment. The result of PI staining showed that the treatment of calycosin H10 could induce the G0/G1 cell cycle arrest in SKOV3 and A2780 cells (Figure 1A). The result of the colony formation assay further supported the suppressive role of calycosin H10 in the tumorigenicity of ovarian carcinoma (Figure 1B). In addition, the result of AnnexinV‐PI staining followed by flow cytometry analysis demonstrated that the calycosin H10 treatment could cause a dose‐dependent induction of cell apoptosis in both SKOV3 and A2780 cells (Figure 1C). In the wound healing assay, the treatment of calycosin H10 reduced the migration ability of ovarian carcinoma (Figure 1D). This result was further supported by the invasion assay, which showed that the treatment of calycosin H10 reduced the number of invasive cells in ovarian carcinoma (Figure 1E). Taken together, our results suggested that calycosin H10 was highly effective for treating ovarian carcinoma.

**TABLE 1:** The lethal concentration 50 (μM) of calycosin and its modified derivative modified in ovarian cancer cells, SKOV3 and A2780.

Cell lines	LC50 (μM)										
	Calycosin	H1	H2	H3	H4	H5	H6	H7	H8	H9	H10
SKOV3	> 32	> 32	> 32	> 32	> 32	> 32	> 32	> 32	> 32	> 32	2.64
A2780	> 32	> 32	> 32	> 32	24.26	12.95	> 32	> 32	21.88	2.10	5.23

### 3.2 Calycosin H10 Controlled the Biological Processes of DNA Damage Response and Cell Proliferation in Ovarian Carcinoma

**FIGURE 2:**
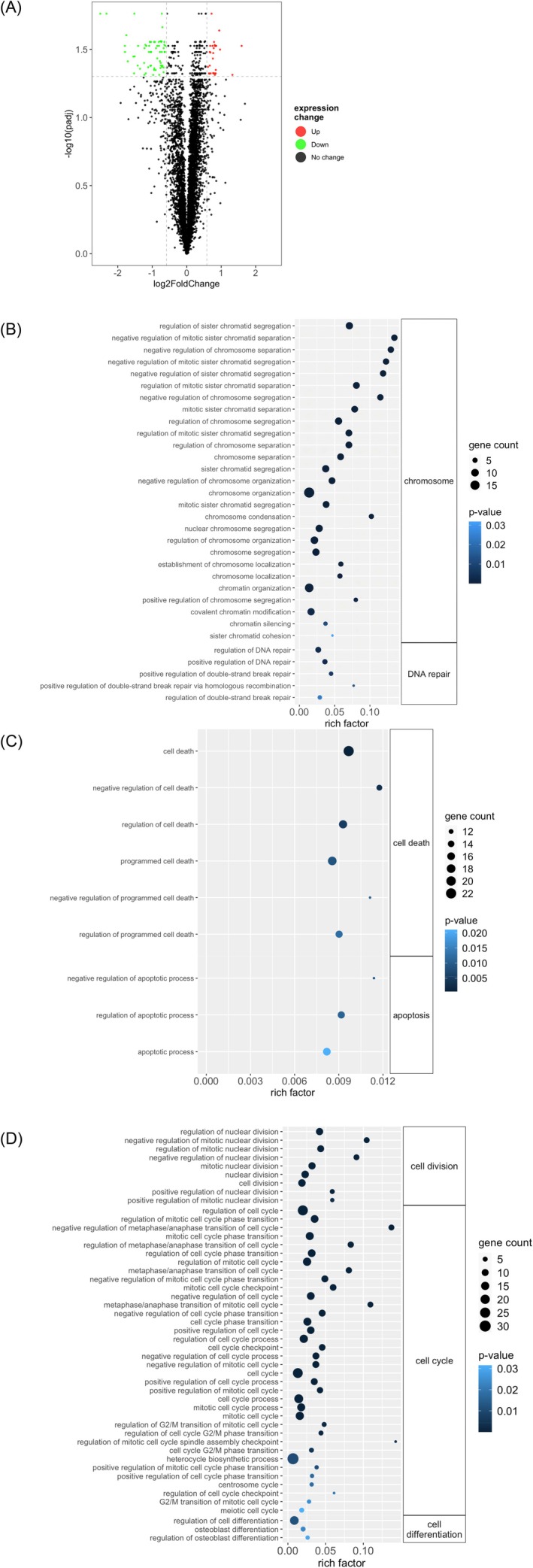
Comparative proteomic analysis delineated the molecular mechanisms underlying the anti‐ovarian carcinogenic effect of calycosin H10. (A) Proteomic analysis comparing the protein profile of calycosin H10 treatment group with that of control group. The green dots represented the downregulated proteins and the red dots represented the upregulated proteins. The gene ontology enrichment analysis of the differentially expressed proteins (DEPs) highlighted their importance in (B) chromosome stability and DNA repair, (C) cell death and cell apoptosis, and (D) cell proliferation. The size of dot represented the number of DEPs. The color of the dots represented the significance of the biological processes.

In order to understand the molecular mechanisms underlying the therapeutic effect of the calycosin H10, comparative proteomic analysis was conducted. When we compared the protein profile of the control group and the calycosin treatment group in SKOV3 cells, we identified 90 differentially expressed proteins (DEPs), including 28 upregulated and 62 downregulated proteins (Figure 2A and Table 2). The DEPs were subjected to gene ontology and KEGG enrichment analysis. Our results showed that the DEPs were involved in the biological processes related to chromosome stability and DNA damage response, such as chromosome segregation and condensation, and double‐strand break repair through the mediation of RAD51AP1, MORF4L1, MORF4L2, USP22, DDX11, and USP1 (Figure 2B). It turned out that the programme cell death, such as cell apoptosis, occurred through the regulation of a cluster of proteins including HELLS, CEBPB, CDKN1B, RRN3, CBX4, SH3KBP1, PLK1, HSPG2, AURKA, IFIT2, MORF4L1, GPNMB, MORF4L2, CTH, NAMPT, PDCD4, TAX1BP1, CDK1, OLR1, DNASE2, SERINC3, and EPHA2 (Figure 2C). In addition, the treatment of calycosin could regulate many biological processes related to the cell cycle through the modulation of G2/M phase transition and cell division (Figure 2D).

**TABLE 2:** Deregulated protein in SKOV3 caused by calycosin H0 treatment.

UniProt ID	Gene symbol	Protein name	Ratio (Treatment/Control)	Adjusted *p*	Up/Down
O00308	WWP2	NEDD4‐like E3 ubiquitin‐protein ligase WWP2	0.64	0.028	Down
O00762	UBE2C	Ubiquitin‐conjugating enzyme E2 C	0.30	0.025	Down
O14817	TSPAN4	Tetraspanin‐4	0.43	0.031	Down
O14965	AURKA	Aurora kinase A	0.53	0.028	Down
O15403	SLC16A6	Monocarboxylate transporter 7	0.43	0.049	Down
O43187	IRAK2	Interleukin‐1 receptor‐associated kinase‐like 2	0.49	0.033	Down
O43493	TGOLN2	Trans‐Golgi network integral membrane protein 2	0.58	0.033	Down
O43736	ITM2A	Integral membrane protein 2A	0.54	0.031	Down
O60637	TSPAN3	Tetraspanin‐3	0.63	0.047	Down
O75496	GMNN	Geminin	0.49	0.028	Down
O94782	USP1	Ubiquitin carboxyl‐terminal hydrolase 1	0.49	0.033	Down
P04114	APOB	Apolipoprotein B‐100	0.20	0.017	Down
P06493	CDK1	Cyclin‐dependent kinase 1	0.61	0.033	Down
P09629	HOXB7	Homeobox protein Hox‐B7	0.60	0.028	Down
P14635	CCNB1	G2/mitotic‐specific cyclin‐B1	0.59	0.040	Down
P15260	IFNGR1	Interferon gamma receptor 1	0.49	0.042	Down
P29317	EPHA2	Ephrin type‐A receptor 2	0.55	0.030	Down
P31350	RRM2	Ribonucleoside‐diphosphate reductase subunit M2	0.57	0.030	Down
P36894	BMPR1A	Bone morphogenetic protein receptor type‐1A	0.53	0.045	Down
P36969	GPX4	Phospholipid hydroperoxide glutathione peroxidase	0.29	0.033	Down
P41229	KDM5C	Lysine‐specific demethylase 5C	0.58	0.049	Down
P49454	CENPF	Centromere protein F	0.59	0.028	Down
P52292	KPNA2	Importin subunit alpha‐1	0.64	0.029	Down
P53350	PLK1	Serine/threonine‐protein kinase PLK1	0.48	0.042	Down
P53816	PLAAT3	Phospholipase A and acyltransferase 3	0.52	0.044	Down
P60520	GABARAPL2	Gamma‐aminobutyric acid receptor‐associated protein‐like 2	0.51	0.049	Down
P63316	TNNC1	Troponin C, slow skeletal and cardiac muscles	0.34	0.036	Down
P78380	OLR1	Oxidized low‐density lipoprotein receptor 1	0.43	0.040	Down
P98179	RBM3	RNA‐binding protein 3	0.35	0.036	Down
Q12834	CDC20	Cell division cycle protein 20 homolog	0.48	0.028	Down
Q13530	SERINC3	Serine incorporator 3	0.48	0.028	Down
Q13772	NCOA4	Nuclear receptor coactivator 4	0.31	0.039	Down
Q14493	SLBP	Histone RNA hairpin‐binding protein	0.59	0.043	Down
Q14596	NBR1	Next to BRCA1 gene 1 protein	0.61	0.017	Down
Q14956	GPNMB	Transmembrane glycoprotein NMB	0.63	0.031	Down
Q15004	PCLAF	PCNA‐associated factor	0.18	0.017	Down
Q15014	MORF4L2	Mortality factor 4‐like protein 2	0.55	0.037	Down
Q15036	SNX17	Sorting nexin‐17	0.44	0.029	Down
Q16763	UBE2S	Ubiquitin‐conjugating enzyme E2 S	0.39	0.044	Down
Q53HL2	CDCA8	Borealin	0.64	0.033	Down
Q86VP1	TAX1BP1	Tax1‐binding protein 1	0.52	0.045	Down
Q8NBI5	SLC43A3	Solute carrier family 43 member 3	0.47	0.033	Down
Q8NC42	RNF149	E3 ubiquitin‐protein ligase RNF149	0.35	0.047	Down
Q8NFJ5	GPRC5A	Retinoic acid‐induced protein 3	0.44	0.047	Down
Q8WUX1	SLC38A5	Sodium‐coupled neutral amino acid transporter 5	0.44	0.030	Down
Q96B01	RAD51AP1	RAD51‐associated protein 1	0.60	0.028	Down
Q96EA4	SPDL1	Protein Spindly	0.65	0.030	Down
Q96FC9	DDX11	ATP‐dependent DNA helicase DDX11	0.46	0.043	Down
Q96HE9	PRR11	Proline‐rich protein 11	0.63	0.043	Down
Q99081	TCF12	Transcription factor 12	0.61	0.042	Down
Q9H492	MAP1LC3A	Microtubule‐associated proteins 1A/1B light chain 3A	0.61	0.042	Down
Q9H7F0	ATP13A3	Polyamine‐transporting ATPase 13A3	0.52	0.028	Down
Q9H7F4	TMEM185B	Transmembrane protein 185B	0.38	0.030	Down
Q9NQS7	INCENP	Inner centromere protein	0.61	0.022	Down
Q9NRZ9	HELLS	Lymphoid‐specific helicase	0.60	0.042	Down
Q9NU53	GINM1	Glycoprotein integral membrane protein 1	0.35	0.017	Down
Q9NV92	NDFIP2	NEDD4 family‐interacting protein 2	0.37	0.042	Down
Q9NYV6	RRN3	RNA polymerase I‐specific transcription initiation factor RRN3	0.64	0.033	Down
Q9UBU8	MORF4L1	Mortality factor 4‐like protein 1	0.57	0.028	Down
Q9ULG6	CCPG1	Cell cycle progression protein 1	0.29	0.030	Down
Q9UPT9	USP22	Ubiquitin carboxyl‐terminal hydrolase 22	0.47	0.030	Down
Q9Y5Z0	BACE2	Beta‐secretase 2	0.58	0.039	Down
O00115	DNASE2	Deoxyribonuclease‐2‐alpha	1.68	0.030	Up
O00257	CBX4	E3 SUMO‐protein ligase CBX4	1.69	0.037	Up
O60488	ACSL4	Long‐chain‐fatty‐acid—CoA ligase 4	1.76	0.049	Up
P05091	ALDH2	Aldehyde dehydrogenase, mitochondrial	1.77	0.030	Up
P09913	IFIT2	Interferon‐induced protein with tetratricopeptide repeats 2	1.70	0.034	Up
P17676	CEBPB	CCAAT/enhancer‐binding protein beta	1.79	0.047	Up
P32929	CTH	Cystathionine gamma‐lyase	1.92	0.023	Up
P43490	NAMPT	Nicotinamide phosphoribosyltransferase	1.79	0.030	Up
P46527	CDKN1B	Cyclin‐dependent kinase inhibitor 1B	1.61	0.030	Up
P46821	MAP1B	Microtubule‐associated protein 1B	1.73	0.028	Up
P49589	CARS1	Cysteine—tRNA ligase, cytoplasmic	1.65	0.017	Up
P98160	HSPG2	Basement membrane‐specific heparan sulfate proteoglycan core protein	1.57	0.047	Up
Q08380	LGALS3BP	Galectin‐3‐binding protein	1.81	0.030	Up
Q13214	SEMA3B	Semaphorin‐3B	1.59	0.033	Up
Q13751	LAMB3	Laminin subunit beta‐3	1.57	0.048	Up
Q15646	OASL	2′‐5′‐oligoadenylate synthase‐like protein	1.70	0.047	Up
Q16270	IGFBP7	Insulin‐like growth factor‐binding protein 7	2.51	0.049	Up
Q53EL6	PDCD4	Programmed cell death protein 4	1.72	0.031	Up
Q8N5F7	NKAP	NF‐kappa‐B‐activating protein	1.61	0.033	Up
Q92522	H1‐10	Histone H1.10	1.59	0.042	Up
Q96B97	SH3KBP1	SH3 domain‐containing kinase‐binding protein 1	1.53	0.043	Up
Q96CM8	ACSF2	Medium‐chain acyl‐CoA ligase ACSF2, mitochondrial	1.95	0.032	Up
Q96EN8	MOCOS	Molybdenum cofactor sulfurase	1.76	0.029	Up
Q96Q06	PLIN4	Perilipin‐4	3.02	0.030	Up
Q9BZQ8	NIBAN1	Protein Niban 1	1.62	0.047	Up
Q9H4F8	SMOC1	SPARC‐related modular calcium‐binding protein 1	1.76	0.047	Up
Q9NR19	ACSS2	Acetyl‐coenzyme A synthetase, cytoplasmic	1.79	0.045	Up
Q9NS39	ADARB2	Double‐stranded RNA‐specific editase B2	1.72	0.047	Up

### 3.3 Calycosin H10 Regulated the Activities of Chromatin and Kinase and Ferroptosis in Ovarian Carcinoma

**FIGURE 3:**
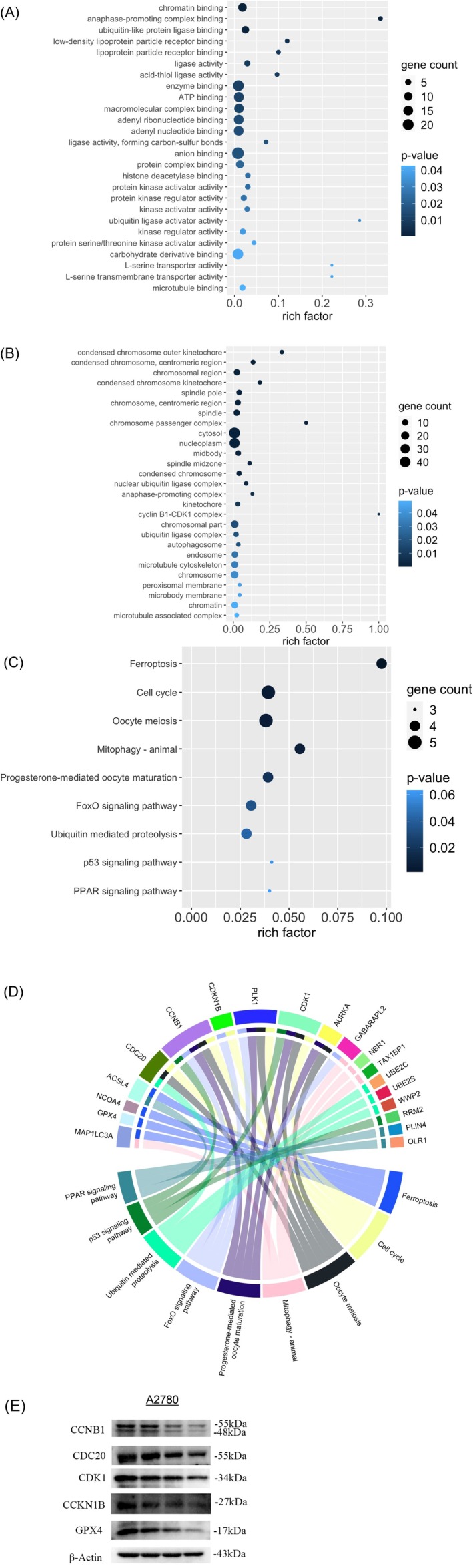
Calycosin H10 targeted the proteins involved in molecular functions and signaling pathways in ovarian carcinoma. (A) Calycosin H10 regulated the molecular functions related to chromatin structure and kinase activity. (B) Gene ontology analysis of the cellular component showed the targets including chromosome structure and microtubule of calycosin H10. (C) Kyoto Encyclopedia of Genes and Genomes enrichment analysis highlighted the regulation of pathways including ferroptosis, ovarian functions, and tumorigenesis in ovarian carcinoma. The size of the dot represented the number of DEPs. The color of the dots represented the significance of the terms. (D) Circos plot showed the involvement of DEPs in the significant signaling pathways. (E) Western blotting was used to validate the result of the proteome in A2780 cells.

The GO analysis of molecular function further highlighted the importance of calycosin in regulating the chromatin structure such as chromatin binding and histone deacetylase binding (Figure 3A). On the other hand, calycosin regulated kinase activity including protein serine/threonine kinase activator activity and protein kinase regulator activity (Figure 3A). Also, microtubule binding activity was controlled by calycosin. Then the GO enrichment analysis of cellular component suggested the compartment of calycosin's functions. Our results showed that the effect of calycosin could be on different chromosome structures, especially condensed chromosome kinetochore, centromeric region of chromosome, and chromatin (Figure 3B). Also, calycosin controlled the proteins involved in microtubule‐related components such as spindle pole, spindle midzone, microtubule cytoskeleton, and microtubule‐associated complex (Figure 3B). In concordance with its effect on the cell cycle, calycosin could regulate the proteins involved in the cyclin B1‐CDK1 complex (Figure 3B). Lastly, the result of the KEGG pathway enrichment analysis demonstrated the control of the cell cycle, ferroptosis, oocyte meiosis, progesterone‐mediated oocyte maturation, and mitophagy by calycosin H10 through the regulation of some pathways such as FoxO signaling pathway, p53 signaling pathway, and PPAR signaling pathway (Figure 3C). The protein cluster including MAP1LC3A, GPX4, NCOA4, ACSL4, CDC20, CCNB1, CDKN1B, PLK1, CDK1, AURKA, GABARAPL2, NBR1, TAX1BP1, UBE2C, UBE2S, WWP2, RRM2, PLIN4, and OLR1 was found to contribute to these pathways (Figure 3D). The result of the proteome was further validated in A2780 cells using Western blotting. Our result showed that the calycosin H10 treatment could lead to a similar reduction of CCNB1, CDC20, CDK1, CDKN1B, and GPX4 in A2780 cells (Figure 3E).

### 3.4 Calycosin H10 Induced Cell Cycle Arrest and Senescence in Ovarian Carcinoma

**FIGURE 4:**
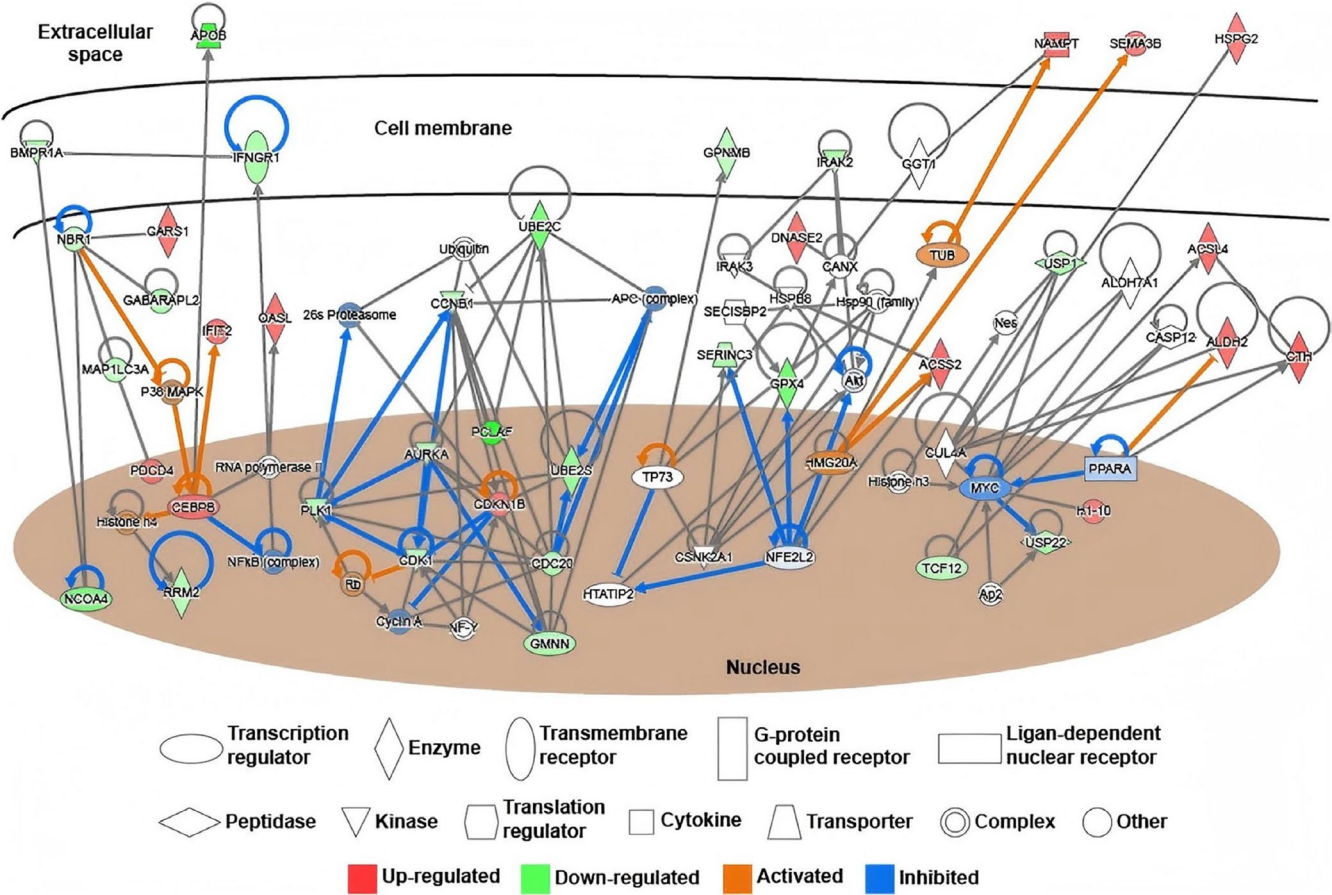
Calycosin H10 controlled a network of proteins involved in the anti‐ovarian carcinoma. Canonical pathway analysis in IPA showed the protein network involved in the calycosin H10‐induced cell cycle arrest and senescence. Different sharps of figures represented different classes of proteins. Red figures represented upregulated proteins, green figures represented downregulated proteins, orange lines represented the predicted activation, and blue lines represented predicted inhibition.

We then conducted Ingenuity Pathway Analysis to delineate the mechanism underlying the anti‐ovarian carcinoma role of calycosin H10. The result of canonical pathways demonstrated that the calycosin H10‐mediated proteins could induce the DNA damage responses, leading to cell cycle arrest in ovarian carcinoma, which was reflected by the negative *z*‐score (Table 3). Also, the treatment of calycosin H10 could induce the senescence of ovarian carcinoma (Table 3). The network construction further showed the involvement of different classes of proteins in the anti‐ovarian carcinoma role of calycosin H10 (Figure 4). It included transporters (APOB and SERINC3), enzymes (CARS1, OASL, RRM2, UBE2C, UBE2S, GPNMB, GPX4, HSPG2, ACSL4, ALDH2, CTH, and ACSS2), kinases (BMPR1A, PLK1, CCNB1, and CDK1), transcription factors (CEBRB, NCOA4, GMNN, and TCF12), peptides (USP1 and USP22), and a cytokine (NAMPT) (Figure 4).

**TABLE 3:** Ingenuity pathway analysis on the differentially expressed proteins in SKOV3 caused by calycosin H0 treatment.

Ingenuity canonical pathways	−log(*p*)	*z*‐score	Molecules
Cell cycle checkpoints	8.25	−2.714	CCNB1, CDC20, CDCA8, CDK1, CDKN1B, CENPF, INCENP, PLK1, SPDL1, UBE2C, UBE2S
Mitotic metaphase and anaphase	7.74	−3.162	CCNB1, CDC20, CDCA8, CDK1, CENPF, INCENP, PLK1, SPDL1, UBE2C, UBE2S
Regulation of mitotic cell cycle	7.41	−2.646	AURKA, CCNB1, CDC20, CDK1, PLK1, UBE2C, UBE2S
Kinetochore metaphase signaling pathway	6.74	−1.89	CCNB1, CDC20, CDCA8, CDK1, INCENP, PLK1, SPDL1
Mitotic prometaphase	6.03	−2.828	CCNB1, CDC20, CDCA8, CDK1, CENPF, INCENP, PLK1, SPDL1
RHO GTPases activate formins	4.87	−2.449	CDC20, CDCA8, CENPF, INCENP, PLK1, SPDL1
TP53 regulates transcription of cell cycle genes	4.48	−1	AURKA, CCNB1, CDK1, CDKN1B
Mitotic roles of polo‐like kinase	3.94	−2	CCNB1, CDC20, CDK1, PLK1
Mitotic G2‐G2/M PHASES	3.05	−2.236	AURKA, CCNB1, CDK1, CENPF, PLK1
ID1 signaling pathway	3.01	−1.342	AURKA, BMPR1A, CDC20, CDKN1B, USP1
Mitotic G1 phase and G1/S transition	2.83	−1	CCNB1, CDK1, CDKN1B, RRM2
Cell Cycle: G2/M DNA damage checkpoint regulation	4.44	2	AURKA, CCNB1, CDK1, PLK1
Ferroptosis signaling pathway	3.88	0.447	ACSL4, CARS1, CTH, GPX4, NCOA4
Senescence‐associated secretory phenotype (SASP)	5.10	0.447	CDKN1B, CEBPB, IGFBP7, UBE2C, UBE2S
Senescence pathway	1.58	2	CCNB1, CDK1, CDKN1B, CEBPB

## 4 Discussion

Limited studies demonstrated the anti‐ovarian carcinogenic role of calycosin, which can be due to its poor water solubility and low bioavailability. In the present report, we aim to modify the calycosin to overcome these limitations and to study the anti‐ovarian tumorigenic roles of the modified calycosin derivatives. Firstly, we obtained 10 modified calycosin derivatives. We found that the calycosin H10 has the highest efficiency for treating ovarian cancer. By using 2 ovarian cancer cell lines SKOV3 and A2780, we conducted a series of in vitro assays to demonstrate the anti‐carcinogenic properties of calycosin H10. Our results showed that calycosin H10 was the most effective derivative for treating ovarian cancer. When we compared our results with a previous report by Zhou's group, it clearly showed that the modified calycosin H10 is more effective in inhibiting the growth of ovarian cancer cells (Zhou et al. 2015).

In the later part of the study, we applied the comparative proteomic analysis to further understand the molecular mechanisms underlying the anti‐ovarian cancer roles of calycosin H10. In the gene ontology analysis of the DEPs, we focused on the biological processes related to the observed functional changes caused by the calycosin H10 treatment. First, we found the regulation of chromosome structure and DNA damage in ovarian cancer by calycosin H10. DNA damage response is important to maintain genomic stability (Petropoulos and Halazonetis 2021). Alteration of this response is closely associated with ovarian cancer development (Ovejero‐Sánchez et al. 2023). Our results highlighted the mediation of DNA damage response by calycosin H10 through the downregulation of a protein cluster such as RAD51AP1, USP1, USP22, and DDX11. Our results showed that calycosin H10 could reduce the expression of RAD51‐associated protein 1 (RAD51AP1). RAD51AP1 is critical for homologous recombination that plays important roles in the repair of DNA double‐strand breaks (Pires et al. 2017). RAD51AP1 was considered a novel biomarker for ovarian cancer diagnosis, and high expression of RAD51AP1 is associated with poorer overall survival (OS) in patients with ovarian cancer (Chudasama et al. 2018). Mechanistically, an in vitro study using ovarian cancer cells demonstrated that RAD51AP1 promoted the progression of ovarian cancer via the TGF‐β/Smad signaling pathway (Zhao et al. 2021).

In addition, our result showed the inhibition of ubiquitin‐specific protease family members USP1 and USP22 caused by the calycosin H10 treatment. The deubiquitinase USP1 is a critical regulator of genome integrity (Coleman et al. 2022). There was only a limited study to show the roles of USP1 in ovarian cancer. For example, Sonego's group demonstrated that inhibition of USP1 increased drug sensitivity and decreased metastatic dissemination in ovarian cancer cells through the control of Snail (Sonego et al. 2019). But there were many studies showing the important roles of USP1 in the carcinogenicity of ovarian cancer. It was reported that inhibition of USP1 activated ER stress through Ubi‐protein aggregation to induce autophagy and apoptosis in HCC (Wang et al. 2022). Both in vitro and in xenograft mouse models suggested that inhibition of USP1 suppressed tumor growth and reduced the drug resistance of prostate cancer cells (Liao et al. 2021). A breast cancer study showed that upregulated USP1 expression in primary breast cancer specimens correlated with metastatic progression and poor prognosis in breast cancer patients; it was further supported by an in vitro study using breast cancer cells that inhibition of USP1 suppressed breast cancer metastasis (Ma et al. 2019). Ubiquitin specific protease 22 (USP22) is considered an oncogene that is commonly upregulated in various malignant tumors and is associated with their poor prognosis (Ji et al. 2015; Feng et al. 2021). For example, USP22 was reported to promote lipogenesis contributing to hepatocellular carcinoma pathogenesis (Xu et al. 2022). In addition, an in vitro study using pancreatic cancer cells showed that downregulation of USP22 reduced cell stemness and enhanced drug sensitivity by inactivating the Wnt/β‐catenin pathway (Li et al. 2022). Another in vitro study using non‐small cell lung cancer demonstrated the importance of USP22 in the malignancy of lung cancer (Sun et al. 2022). So, calycosin H10‐inhibited USP1 and USP22 should be promising targets to induce cancer cell death (Woo et al. 2022).

We then conducted KEGG pathway enrichment analysis to further delineate the signaling pathways involved in the anti‐ovarian carcinogenic roles of calycosin H10. Our results highlighted the regulation of ferroptosis. Ferroptosis is considered a programmed metabolic cell death that is driven by iron‐dependent phospholipid peroxidation (Jiang et al. 2021). In the past decade, targeting ferroptosis has been used as a novel approach for treating cancer (Zhang, Kong, et al. 2022), especially for cancer immunotherapy (Zhao et al. 2022). In our analysis, we found that calycosin H10 regulated a cluster of ferroptosis‐related genes such as ACSL4, GPX4, and NCOA4. Acyl‐CoA Synthetase Long Chain Family Member 4 (ACSL4) is responsible for encoding long‐chain fatty‐acid‐coenzyme A ligase (Piccini et al. 1998). A study using a genome‐wide CRISPR‐based genetic screen and microarray analysis of ferroptosis‐resistant cell lines showed that ACSL4 is an essential component for ferroptosis execution (Doll et al. 2017). A study using Oncomine and The Cancer Genome Atlas (TCGA) databases together with in vitro clear cell renal cell carcinoma showed that suppression of ACSL4 increased ferroptosis resistance, whereas overexpression of ACSL4 could restore ferroptosis sensitization in cancer cells (Guo 2022). Glutathione Peroxidase 4 (GPX4) is responsible for lipid peroxidation (Xu et al. 2021). Genetic studies using both in vitro and in vivo mice models suggested that GPX4 is a key regulator of ferroptosis (Seibt et al. 2019). Inhibition of GPX4 function could lead to the induction of ferroptosis and increased cellular ferroptosis sensitivity (Forcina and Dixon 2019; Ingold et al. 2018). In cancer research, targeting GPX4 has been used to induce ferroptosis for tackling cancer resilience (Lee and Roh 2023). For instance, a study of sensitivity profiling in 177 cancer cell lines revealed that diffuse large B cell lymphomas and renal cell carcinomas were susceptible to GPX4‐regulated ferroptosis (Yang et al. 2014). In vivo experiments of triple negative breast cancer showed that inhibition of GPX4 enhanced the anticancer effect of gefitinib through promoting ferroptosis (Song et al. 2020). In addition, targeting GPX4 was a therapeutic approach in clear‐cell carcinomas through conferring sensitivity to ferroptosis (Zou et al. 2019). Nuclear receptor coactivator 4 (NCOA4), a cargo receptor, is responsible for ferritin turnover by expediting ferritinophagy, so modulating NCOA4‐mediated ferritinophagic flux would alter sensitivity to ferroptosis (Santana‐Codina et al. 2021). It was reported that NCOA4 depletion weakened ferroptosis, leading to unfavorable outcomes and defective immune cell infiltration in clear cell renal carcinoma (Mou et al. 2021). On the other hand, induction of NCOA4 expression increased ferroptosis in liver cancer cells and glioblastoma cells (Yang et al. 2023; Zhang, Kong, et al. 2021).

Our result of KEGG pathway analysis also highlighted the regulation of 2 important cancer‐related signaling pathways including FoxO signaling and p53 signaling pathways by calycosin H10. Forkhead box O (FoxO) signaling was suggested to play roles in cell fate decisions and act as a tumor suppressor in a wide range of cancers (Farhan et al. 2017). Because it is a crucial downstream effector of the PI3K/Akt pathway, which plays roles in DNA damage response and apoptosis in ovarian cancer (Shi et al. 2021). In ovarian cancer, the reduced expression of FOXO was associated with resistance to anti‐cancer agents and with reduced efficacy of drug combinations (Beretta et al. 2019). Repressed FoxO transcription factors could allow cell cycle progression in ovarian cancer (Shao et al. 2016). Furthermore, our analysis suggested the control of p53 signaling and cell cycle by calycosin H10 through the reduction of key proteins including CCNB1 and CDK1. P53 signaling is one of the most famous signaling pathways involved in the carcinogenesis of many cancers (Vousden and Lane 2007). A study using orthotopic xenograft models of ovarian clear cell carcinoma showed that p53 degradation suppressed ferroptosis and facilitated ovarian cancer tumorigenesis (Wang et al. 2023). Cyclin B1 (CCNB1) is regarded as an oncogene in multiple tumors (Xie et al. 2019). A report using three microarray datasets of ovarian carcinoma showed that ovarian cancer patients with higher CCNB1 expression were associated with poorer overall survival and progression‐free survival (Yang et al. 2022). Another cell cycle regulator, cyclin‐dependent kinase 1 (CDK1), was also involved in the progression of multiple types of cancer, including colorectal cancer, liver cancer, and lung cancer. The upregulation of CDK1 was associated with reduced survival time for these diseases (Li et al. 2020). An immunohistochemical analysis of 119 human ovarian cancer samples showed that the expression of CDK1 is a prognostic factor in epithelial ovarian cancer, and knockdown of CDK1 promoted apoptosis and increased the sensitivity of epithelial ovarian cancer to chemotherapy drugs (Xi et al. 2015). In addition, the use of a CDK1 inhibitor could inhibit the growth of ovarian cancer (Yang et al. 2016).

In conclusion, our results suggested that the modified calycosin derivative, calycosin H10, is more efficient to treat ovarian carcinoma. The proteomic analysis further delineated that the anti‐ovarian carcinogenic mechanisms of calycosin H10 were through the regulation of ferroptosis and many signaling pathways involved in cell cycle arrest, such as p53 signaling and FOXO signaling. The data of the present study provided a novel insight into the anti‐ovarian carcinogenic roles of calycosin H10, suggesting the possible use of calycosin H10 for combination chemotherapy. But further preclinical study is still needed to confirm the safety of calycosin H10 for chemotherapy before clinical use.

## Author Contributions

**Fuhong Yang:** data curation (equal), methodology (equal), validation (equal), writing – original draft (equal). **Xin Li:** data curation (equal), formal analysis (equal), methodology (equal), writing – original draft (equal). **Hanchi Gao:** data curation (equal), formal analysis (equal), methodology (equal), writing – original draft (equal). **Pei Yao:** data curation (equal), investigation (equal). **Xian Qin:** data curation (equal), formal analysis (equal). **Xiao Lin:** data curation (equal), formal analysis (equal). **Keng Po Lai:** conceptualization (equal), investigation (equal), supervision (equal), writing – original draft (equal), writing – review and editing (equal). **Jing Tian:** conceptualization (equal), funding acquisition (equal), project administration (equal), supervision (equal), writing – original draft (equal), writing – review and editing (equal). **Jian Chen:** conceptualization (equal), funding acquisition (equal), project administration (equal), supervision (equal), writing – original draft (equal), writing – review and editing (equal).

## Funding

This research is respectively supported by the National Natural Science Foundation of China (81973574, 82060736, 82160282), Natural Science Foundation of Guangxi Autonomous Region (2025GXNSFAA069839, 2019GXNSFFA245001, 2017GXNSFDA198019, 2018GXN).

## Ethics Statement

The authors have nothing to report.

## Conflicts of Interest

The authors declare no conflicts of interest.

## Supporting information


**Figure S1:** fsn371338‐sup‐0001‐FigureS1.docx.

## Data Availability

Data available on request from the authors.
