# Correction to “Biochemical and Comparative Proteomic Analyses Delineate the Anti‐Ovarian Carcinogenic Roles of Modified Calycosin”

**DOI:** 10.1002/fsn3.71810

**Published:** 2026-04-17

Yang F, Li X, Gao H, Yao P, Qin X, Lin X, Lai KP, Tian J, Chen J. Biochemical and Comparative Proteomic Analyses Delineate the Anti‐Ovarian Carcinogenic Roles of Modified Calycosin. *Food Science & Nutrition* 2026;14(1): e71338.

In the Funding section, the funding source, “Guangxi Science and Technology Base and Talent Special Project (No. AD23026092),” was inadvertently omitted. The complete Funding statement should read as follows:

“This research is supported by the Guangxi Science and Technology Base and Talent Special Project (No. AD23026092), the Natural Science Foundation of Guangxi Autonomous Region (2025GXNSFAA069839, 2019GXNSFFA245001, 2017GXNSFDA198019), the National Natural Science Foundation of China (81973574, 82060736, 82160282), and the Bagui Top‐notch Talent Program.”

We apologize for this error.

