# Supplementary figures and images for: Biochemical and Comparative Proteomic Analyses Delineate the Anti‐Ovarian Carcinogenic Roles of Modified Calycosin
Source: Food Sci Nutr. 2026 Jan 14;14(1):e71338. doi: 10.1002/fsn3.71338 (PMC12802411; doi:10.1002/fsn3.71338)

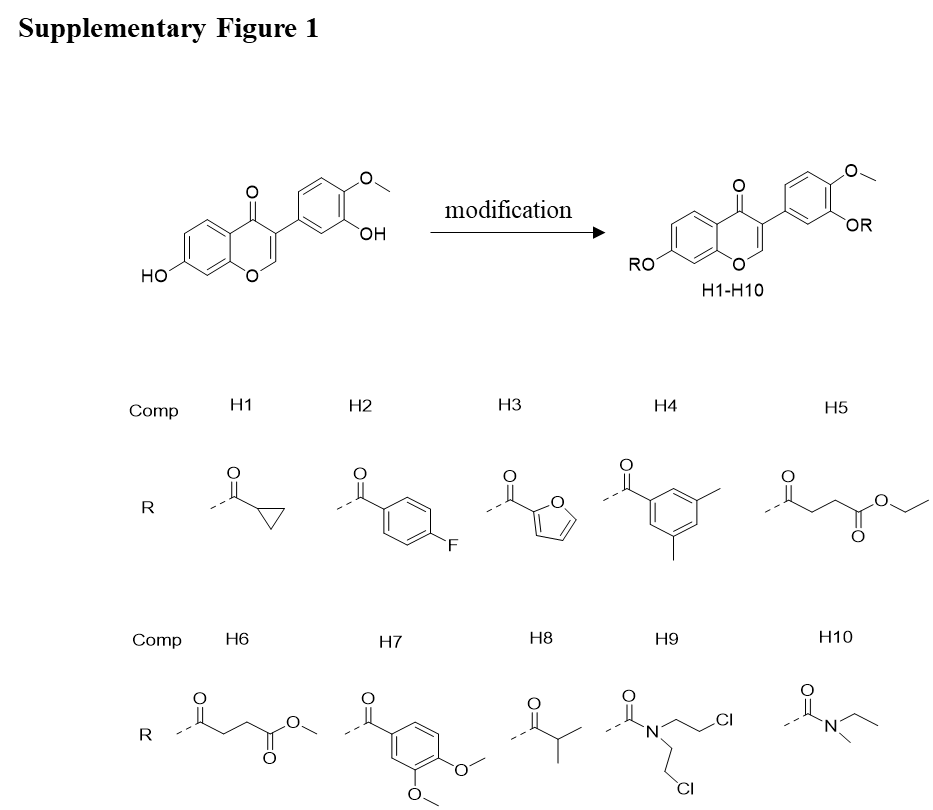


Figure S1: Structural modification of calycosin and its derivatives (H1-H10)

Supplement: Supplementary file 1 — Figure S1: fsn371338‐sup‐0001‐FigureS1.docx. [file FSN3-14-e71338-s001.docx]
